# Risk factors for parastomal hernia following abdominoperineal resection

**DOI:** 10.3389/fonc.2025.1692769

**Published:** 2025-10-29

**Authors:** An Shang, Liping Li, Ge He, Donggui Zhuge, Pengcheng Yu, Junyi Xu

**Affiliations:** ^1^ Department of the General Surgery, The Fourth Hospital of Guangxi Medical University, Liuzhou, Guangxi, China; ^2^ Department of Pneumology, The Fourth Hospital of Guangxi Medical University, Liuzhou, Guangxi, China

**Keywords:** rectal cancer, APR, PSH, miles operation, risk factors

## Abstract

**Objective:**

Parastomal hernia (PSH) is a common complication after stoma construction, particularly in patients with colostomy, with an incidence of up to 50%. The primary objective of the present study was to explore the clinical and radiological risk factors for the development of PSH in patients who underwent abdominoperineal resection, thereby helping surgeons identify high-risk patients and select appropriate individualized follow-up and treatment strategies.

**Methods:**

All consecutive patients who underwent abdominoperineal resection (APR) in the left lower abdomen were considered for inclusion in the present study according to the inclusion and exclusion criteria from January 2017 to May 2022. The follow-up period of selected patients was at least 1 year. A total of 18 potential risk factors for PSH were evaluated. Univariate and multivariate binary logistic regression analyses were performed to identify factors significantly associated with PSH development. The Kaplan-Meier method was used to evaluate the association between risk factors and the cumulative incidence of PSH.

**Results:**

In our study, the incidence of PSH was 44.4%. In the final multivariate analysis, we identified three independent risk factors, including thickness of rectus abdominis, SAT percentage and colostomy surface area. In addition, we found that high SAT percentage (>median) and large colostomy surface area (>median) were associated with a higher three-year incidence rate than the control group (56.7% vs. 21.5% and 47.3% vs. 33.6%). However, the conclusion was opposite when the thickness of rectus abdominis was analyzed (36.7% vs. 46.2%).

**Conclusion:**

In the present study, we found that the thickness of the rectus abdominis, the SAT percentage, and the colostomy surface area were significantly associated with the development of PSH, which may be potential predictors for PSH. In particular, our study reported the potential predictive value of the thickness of rectus abdominis for the development of PSH for the first time.

## Introduction

Parastomal hernia(PSH), a type of incisional hernia, is an abnormal protrusion through the abdominal wall defect created during the placement of an ileostomy, colostomy, or ileal conduit stoma (1). It is a common complication after stoma construction, especially in patients with colostomy, with an incidence of up to 50% (2, 3). Studies reported that PSH has a negative impact on quality of life (QOL) and body image experience (4, 5), containing pain (35%) and problems with stoma appliance (28%), often resulting in leakage (27%) (6). Many stoma-related problems will occur following stoma appliance leakage, such as peristomal dermatitis, unpleasant odor, and soilage of clothes. Additionally and importantly, approximately 30% PSH patients underwent surgical repair eventually (7) due to the occurrence of complications (such as strangulation, incarceration or obstruction), discomfort and problems with the fitting and function of the appliance (8). Unfortunately, even applying mesh, the recurrence rate of PSH surgery is still as high as 5-76% (7, 9). As such, PSH greatly increases healthcare consumption, work disability, and costs to society.

In recent years, there has been a growing number of studies demonstrating that extraperitoneal colostomy may reduce the incidence of PSH in patients after end colostomy (10, 11). However, one of the complications with extraperitoneal colostomy is a strangulated internal hernia, which is a serious threat to the life safety of the patients (12). Although the incidence of strangulated internal hernia is not high, the clinical application is limited due to the postoperative complications. Therefore, detecting the risk factors for PSH has an important role in reducing the incidence postoperatively, because surgeons are more likely to perform extraperitoneal colostomy in patients with a high risk of PSH due to the limitations of extraperitoneal colostomy (12).

Parastomal hernia is complex in etiology; several studies suggest that higher BMI, older age, female, larger aperture size, and a larger waist circumference were independent risk factors for PSH (13–15). However, these clinical parameters are insufficient for accurate prediction of PSH development. For patients diagnosed with rectal cancers, Computed Tomography (CT) examination was routinely performed to assess carcinoma characteristics, thus tailoring the operation protocol. Many body metrics could be obtained from preoperative CT images, such as thickness of subcutaneous abdominal fat, subcutaneous fat content of abdomen, visceral adipose tissue content of abdomen, and et.al, which may be associated with the development of PSH postoperatively. To date, few studies exploring the predictive value of radiologic data for PSH have been published. Takashi et al. reported that subcutaneous fat area is significantly associated with the development of PSH after colostomy (16). In their research, patients were divided into two groups according to the receiver operating characteristic (ROC) curve of variables. However, the predictive value of variables was not tested by the validation cohort, thus the reliability and reproducibility of the predictor were unknown. Jan Pieter et.al (17), also analyzed the association between radiologic predictors and PSH, where only the diameter of rectus muscle diastasis was significantly associated with PSH, which may be related to the relatively small sample size. The use of CT in predicting PSH development remains controversial.

The primary objective of the present study was to explore clinical and radiological risk factors for the development of PSH in patients who underwent abdominoperineal resection, thus helping surgeons screen out high-risk patients and select appropriate individualized follow-up and treatment strategies.

## Materials and methods

### Patients

All consecutive patients who underwent abdominoperineal resection (APR) in the left lower abdomen between January 2017 and May 2022 were considered for inclusion in the present study based on the inclusion and exclusion criteria. These patients were retrieved from the electronic medical records of the Fourth Hospital of Guangxi Medical University, and their data were analyzed retrospectively. The present research was approved by the Ethics Committee of the Fourth Hospital of Guangxi Medical University and carried out in accordance with the Declaration of Helsinki. The ethical review number is KY2025587.

### Inclusion and exclusion criteria

Inclusion criteria: 1) age between 18 and 75 years old, 2) patients diagnosed with low rectal or anal canal carcinoma, 3) patients who underwent APR and the stoma was sited at the left lower abdomen, 4) computerized tomography (CT) was performed within 2 months preoperatively. Exclusion criteria: 1) patients who underwent emergency surgery, 2) Incomplete baseline data, 3) lack of complete one-year follow-up data post-surgery.

### Surgical techniques

Each patient included in the present study received a standard construction of a permanent end colostomy at the left lower abdomen, performed by a specialized colorectal surgeon. The operative procedure was as follows: A circular incision would be made at the marked site preoperatively, and the abdominal cavity would be entered through a cross-shaped incision along the fascia and peritoneum. After that, the bowel would be taken out through the circular incision and rectus muscle and fixated to the fascia and peritoneum with a 1/0 silk suture. The stoma has to protrude 1 to 2 cm above the skin, fixed with size 3–0 absorbable sutures to the skin (18).

### Definition of PSH and follow-up

PSH was diagnosed by physical examination and abdominal CT following the 2023 European Society of Hernia guidelines (19), which was defined as an incisional hernia occurring at or adjacent to the end colostomy, with the hernia sac containing the omentum, small bowel, colon, or a combination thereof (**Figure 2**). Postoperative examinations were performed according to the international guidelines, including physical examinations every 3 months and abdominal CT every 6 months during the first 2 years, followed by examinations every 12 months for the subsequent 5 years. The final follow-up date for all cases was June 30, 2025. The follow-up period for included patients was at least 1 year, as most PSHs occur early after surgery (20).

### Variables included in the present research

A total of 18 potential risk factors for PSH were evaluated. These included clinical data: gender, age, Body Mass Index (BMI), diabetes, chronic obstructive pulmonary disease (COPD), neoadjuvant therapy, preoperative hemoglobin level, preoperative albumin level, American Society of Anesthesiologists (ASA) classification, operative time, intra-operative blood loss and prior abdominal surgery; radiologic data: thickness of subcutaneous adipose tissue (SAT), thickness of rectus abdominis, SAT percentage, visceral adipose tissue (VAT) percentage, Skeletal muscle rate and colostomy surface area.

### Radiologic measurements

**Figure 1:**
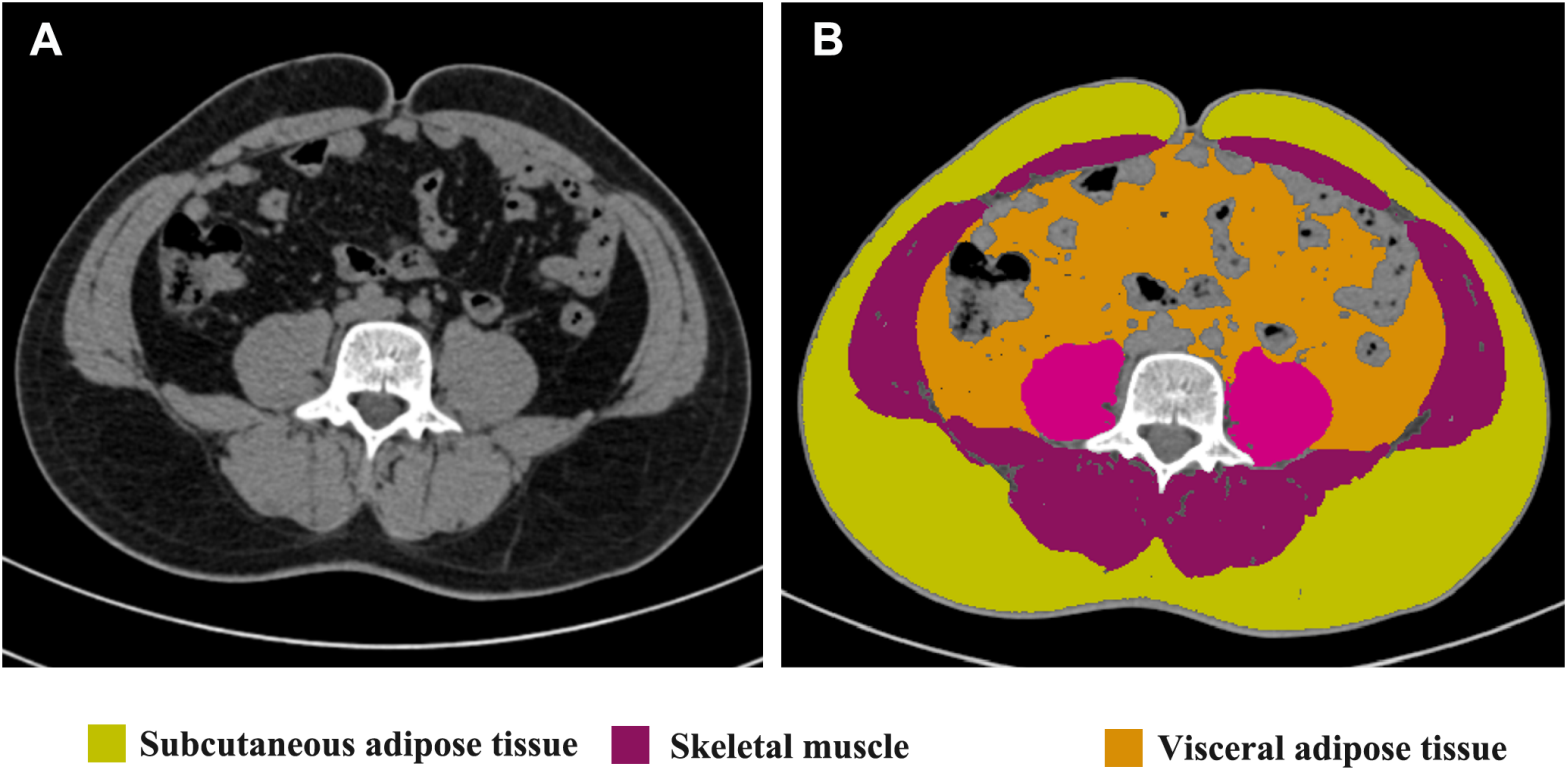
Radiological measurements on the CT scans. Unprocessed CT slice at the level of the umbilicus performed preoperatively is shown in **(A)**. Body metrics evaluation performed using slice‐O‐matic image analysis software is shown in **(B)**. Extraperitoneal skeletal muscles (purple), subcutaneous adipose tissue (yellow) and visceral adipose tissue (brown) are seen.

The measurements of thickness of subcutaneous adipose tissue (SAT), thickness of rectus abdominis, SAT percentage, visceral adipose tissue (VAT) percentage, and Skeletal muscle rate were performed on an axial CT slice at the level of the umbilicus because the stoma is usually placed about the level of the umbilicus (21). Images in Digital Imaging and Communications in Medicine (DICOM) format at the level of the umbilicus from preoperative CT scans were analyzed using slice‐O‐matic image analysis software (version 5.0, Rev-9, Canada), as shown in **Figure 1**. Standard, validated CT Hounsfield unit thresholds were adopted in the present study for the segmentation, ranging from −29 to 150 for skeletal muscle (SM), −190 to −30 for SAT, and −150 to −50 for VAT (22).

**Figure 2:**
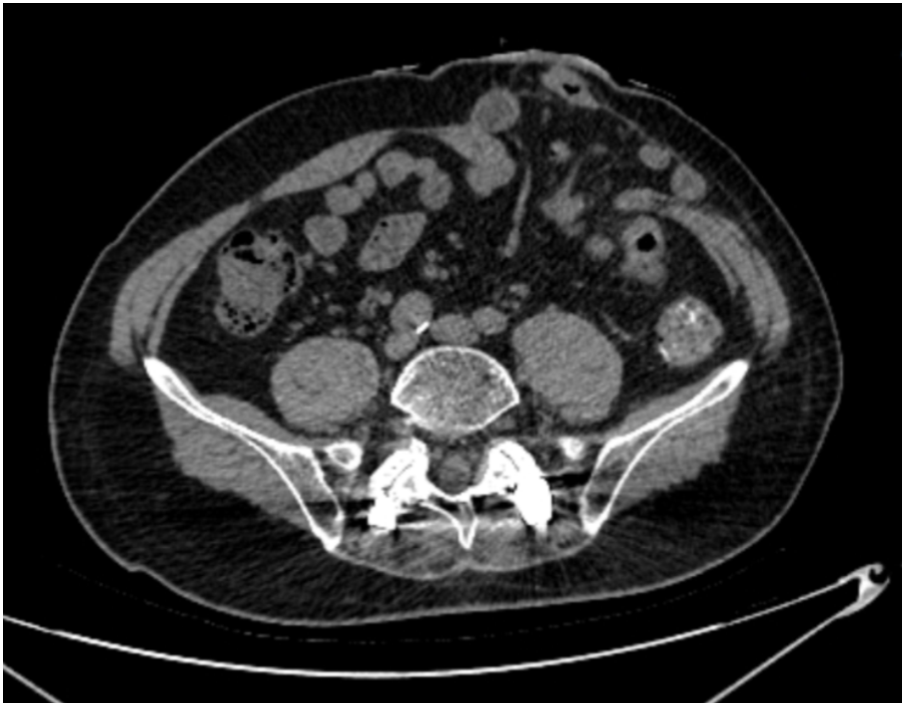
Abdominal CT scan shows herniation of small-bowel loop into PSH.

A vertical line was made at the midpoint of the left rectus abdominis muscle on the axial CT slice to measure the thickness of SAT (mm) and rectus abdominis (mm), in which SAT thickness was defined as the distance between the skin and the external surface of the rectus abdominis muscle. The SAT percentage was calculated by dividing the subcutaneous fat area by the total abdominal section surface area on the axial CT slice. Total abdominal section surface area was calculated from the abdominal anteroposterior diameter and abdominal transverse diameter measured from a transverse section of the abdomen at the level of the umbilicus, where the area= π × [(0.5 × abdominal anteroposterior diameter) × (0.5 × abdominal transverse diameter)]. Similarly, SM percentage was calculated by dividing the skeletal muscle area by the total abdominal section surface area. The VAT percentage was calculated by dividing visceral adipose tissue area by total abdominal cavity section surface area, which was calculated with the following formula: π × [(0.5 × left-to-right distance of the abdominal cavity) × (0.5 × anteroposterior distance of the abdominal cavity)]. The stoma area (square centimeters), defined as the total surface area of the abdominal wall defect at the end colostomy site, was calculated with the following formula: π × [(0.5 × left-to-right distance) × (0.5 × cranial-to-caudal distance)] because the stoma was almost always elliptic-shaped.

Horizontal and vertical diameters of the stoma were measured using the first postoperative abdominal CT scan.

### Statistical analysis

For the patients’ clinical data, continuous variables were expressed as the mean ± standard deviation, and the categorical variables were presented as frequencies. Univariate analysis was performed with univariate binary logistic regression to identify factors significantly associated with PSH development. To identify independent factors of PSH, we performed multivariate analysis using binary logistic regression analysis, in which all factors with p values of 0.10 or less at univariate analysis were included. Later, the Kaplan-Meier method was used to evaluate the association between risk factors and the cumulative incidence of PSH. Finally, statistical analyses were performed using SPSS software, version 29.0.2.0 (IBM Corporation).

## Results

**Figure 3:**
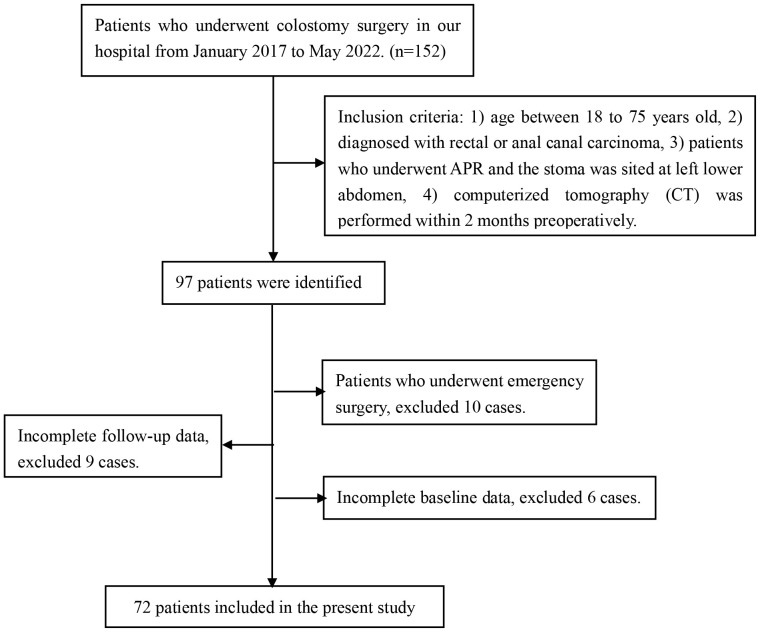
Flowchart of case data collection.

From January 2017 to May 2022, a total of 152 patients who underwent colostomy surgery in the Fourth Affiliated Hospital of Guangxi Medical University were collected, where 97 cases were identified to be further assessed according to the inclusion criteria. After that, 25 cases were excluded when performing the exclusion criteria, in which 10 cases were excluded due to emergency surgery, 9 cases were excluded due to incomplete follow-up data, and 6 cases were excluded due to incomplete baseline data. Eventually, 72 patients were included in the present research, as shown in **Figure 3**.

### Patient characteristics

In the present research, 72 patients were assessed for eligibility, containing 46 men (63.9%) and 26 women (36.1%) with a mean (SD) age of 58.6 (10.74) years. The mean body mass index (BMI) was 22.54 kg/m^2^ with a standard deviation of 2.79. The percentage of patients diagnosed with chronic obstructive pulmonary disease and diabetes mellitus was 6.9% and 4.2%, respectively. In three patients (4.2%), the ASA classification was stage III. Five in 72 patients (6.9%) had a history of previous abdominal surgery. Other baseline characteristics are reported in **Table 1**.

**Table 1:** Baseline characteristics of patients.

Characteristic	Value
Patients, No (%)	
Total no.	72
Men	46 (63.9)
Women	26 (36.1)
Age, mean (SD), y	58.56 (10.74)
BMI, mean (SD), kg/m2	22.54 (2.79)
Diabetes mellitus, No (%)	3 (4.2)
Chronic obstructive pulmonary disease, No (%)	5 (6.9)
Neoadjuvant treatment, No (%)	9 (12.5)
Preoperative laboratory results	
Hemoglobin, mean (SD), g/L	128.99 (11.90)
Albumin, mean (SD), g/L	40.04 (2.89)
ASA, No (%)	
II	69 (95.8)
III	3 (4.2)
Operation time, mean (SD), min	237.21 (55.39)
Amount of intraoperation bleeding, mean (SD), ml	131.18 (91.73)
Prior abdominal surgery, No (%)	5 (6.9)

BMI, body Mass Index; SD, standard deviation; ASA, American Society of Anesthesiologists.

### Radiologic characteristics

The mean thickness of subcutaneous abdominal fat was 15.86 mm (SD, 8.18), the mean thickness of rectus abdominis was 8.67 mm (SD, 2.37), the mean subcutaneous fat content of abdomen was 124.06 cm^2^ (SD, 66.66), the mean visceral adipose tissue content of abdomen was 82.68 cm^2^ (SD, 51.94), the mean skeletal muscle content of abdomen was 90.94 cm^2^ (SD, 20.14), the mean subcutaneous fat percentage was 28.25% (SD, 12.34%), the mean visceral adipose tissue percentage was 40.47% (SD, 19.73), the mean skeletal muscle rate was 22.54% (SD, 6.52) and the mean colostomy surface area was 6.99 cm^2^ (SD, 1.70). A total of 32 patients (44.4%) had a PSH at follow-up CT during a median follow-up time of 9.3 months. Other body indices are reported in **Table 2**.

**Table 2:** CT findings of the included patients.

CT measurement results	Value, mean (SD)
Thickness of subcutaneous abdominal fat, mm	15.86 (8.18)
Thickness of rectus abdominis, mm	8.67 (2.37)
Left-to-right diameter of abdomen, cm	29.20 (2.52)
Front and rear diameter of abdomen, cm	18.18 (2.82)
Cross-sectional surface area of the abdomen, cm^2^	420.86 (90.80)
Left-to-right diameter of abdominal cavity, cm	22.97 (2.08)
Front and rear diameter of abdominal cavity, cm	10.68 (2.16)
Cross-sectional surface area of the abdominal cavity, cm^2^	194.95 (54.74)
Subcutaneous fat content of abdomen, cm^2^	124.06 (66.66)
Visceral adipose tissue content of abdomen, cm^2^	82.68 (51.94)
Skeletal muscle content of abdomen, cm^2^	90.94 (20.14)
Subcutaneous fat rate, %	28.25 (12.34)
Visceral adipose tissue rate, %	40.47 (19.73)
Skeletal muscle rate, %	22.54 (6.52)
Left-to-right diameter of colostomy, cm	2.70 (0.44)
Craniocaudal diameter of colostomy, cm	3.27 (0.44)
Colostomy surface area, cm^2^	6.99 (1.70)
Parastomal hernia, No (%)	32 (44.4)

CT, computerized tomography, SD, standard deviation.

### Univariate and multivariate analyses

To determine independent risk factors for the development of PSH, univariate and multivariate analyses were performed using logistic regression models **Table 3**. The risk variables included gender, age, BMI, diabetes mellitus, COPD, neoadjuvant treatment, preoperative hemoglobin, preoperative albumin, ASA, operation time, amount of intraoperative bleeding, prior abdominal surgery, thickness of SAT, thickness of rectus abdominis, SAT percentage, VAT percentage, skeletal muscle percentage, and colostomy surface area.

**Table 3:** Univariate and multivariate analysis of predictive factors of parastomal hernia development.

Characteristic	Univariate analysis		Multivariate analysis	
	OR (95% CI)	P Value	OR (95% CI)	P Value
Gender, female	9.44(3.01-29.09)	<0.001	2.92(0.30-28.40)	0.36
Age	1.02(0.98-1.67)	0.40	–	–
BMI, >25kg/m^2^	1.85(0.60-5.67)	0.29	–	–
Diabetes mellitus	–	–	–	–
COPD	0.82(0.13-5.24)	0.84	–	–
Neoadjuvant treatment	0.31(0.06-1.63)	0.17	–	–
Preoperative hemoglobin, g/L	0.98(0.94-1.02)	0.27	–	–
Preoperative albumin, g/L	0.99(0.84-1.16)	0.88	–	–
ASA, III	2.60(0.23-30.05)	0.44	–	–
Operation time	0.99(0.98-1.00)	0.35	–	–
Amount of intraoperation bleeding	1.00(0.99-1.01)	0.32	–	–
Prior abdominal surgery	1.97(0.31-12.54)	0.48	–	–
Thickness of SAT, mm	1.18(1.01-1.30)	<0.001	1.06(0.90-1.25)	0.46
Thickness of rectus abdominis, mm	0.63(0.48-0.83)	<0.001	0.64(0.42-0.98)	0.04
SAT rate, %	1.14(1.07-1.21)	<0.001	1.22(1.05-1.41)	0.009
VAT rate, %	1.07(1.03-1.10)	<0.001	1.04(0.97-1.13)	0.28
Skeletal muscle rate, %	0.84(0.76-0.93)	<0.001	1.15(0.91-1.46)	0.23
Colostomy surface area, cm^2^	1.57(1.12-2.19)	0.006	2.76(1.42-5.34)	0.003

BMI, body Mass Index; COPD, chronic obstructive pulmonary disease; ASA, American Society of Anesthesiologists; SAT, subcutaneous adipose tissue; VAT, visceral adipose tissue; OR, odds ratio; CI, confidence interval.

In the univariate analysis, female (OR 9.44, 95%CI 3.01-29.09, P < 0.001), thickness of SAT (OR 1.18, 95%CI 1.01-1.30, P<0.001), thickness of rectus abdominis (OR 0.63, 95%CI 0.48-0.83, P < 0.001, SAT rate (OR 1.14, 95%CI 1.07-1.21, P < 0.001, VAT rate (OR 1.07, 95%CI 1.03-1.10, P < 0.001, skeletal muscle rate (OR 0.84, 95%CI 0.76-0.93, P < 0.001) and colostomy surface area (OR 1.57, 95%CI 1.12-2.19, P = 0.006) were significant associated with the development of PSH, while age, BMI, COPD and prior abdominal surgery were not. In the final multivariate logistic regression model, thickness of rectus abdominis played a protective role in the development of PSH (OR 0.64, 95%CI 0.42-0.98, P = 0.04), however, SAT rate and colostomy surface area independent of other factors promoted the development of PSH (OR 1.22, 95%CI 1.05-1.41, P = 0.009; OR 2.76, 95%CI 1.42-5.34, P = 0.003).

### K‐M cumulative incidence curves

**Figure 4:**
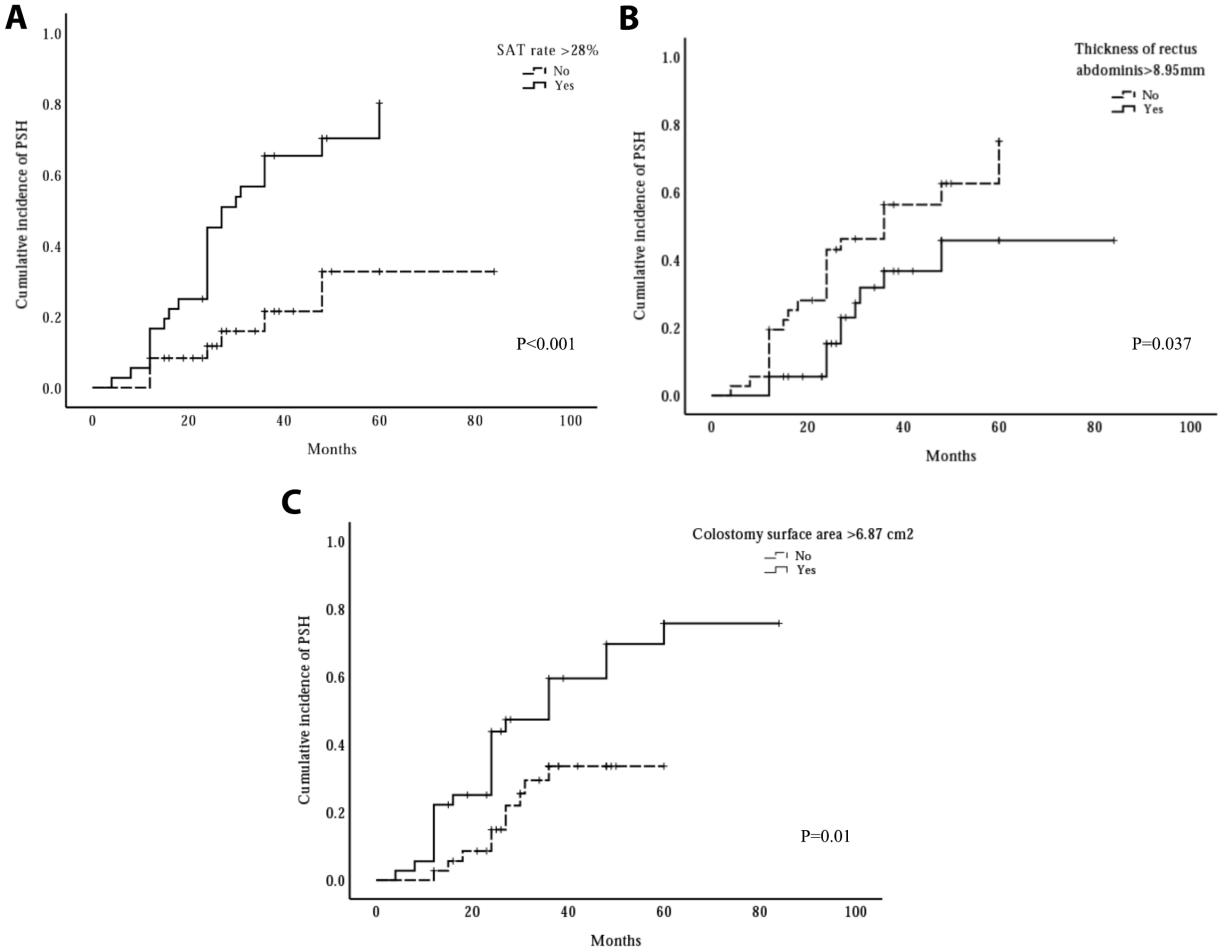
The influence of three risk factors on cumulative incidence of parastomal hernia (PSH). The cumulative incidence rate significantly increased when subcutaneous adipose tissue (SAT) rate >28% **(A)** or colostomy surface area >6.87cm^2^ **(C)**, whereas, the cumulative incidence rate significantly decreased when the thickness of rectus abdominis >8.95 mm **(B)**.

In the present study, 32 patients developed PSH. To further analyze the relationship between the thickness of the rectus abdominis, the SAT rate, and colostomy surface area and cumulative incidence, Kaplan-Meier analyses were performed, as shown in **Figure 4**. Kaplan–Meier analysis indicated that the high SAT percentage (> median) group had a higher cumulative incidence rate of PSH than the low SAT percentage group (56.7% vs. 21.5%, P < 0.001, Kaplan-Meier log-rank). Likewise, the 3-year cumulative incidence rate of PSH in the large colostomy surface area group (> median) was higher than the cumulative incidence rate in the small colostomy surface area group (47.3% vs. 33.6%, P = 0.01, Kaplan-Meier log-rank). However, the high-score group of thickness of rectus abdominis (> median) performed a lower 3-year cumulative incidence rate of PSH than the low-score group (36.7% vs. 46.2%, P = 0.037, Kaplan-Meier log-rank).

## Discussion

The incidence of rectal cancer is increasing globally, especially for younger demographics (23). Although the rectal cancer sphincter preservation rate has been improved following advancements in surgical techniques and the advent of neoadjuvant therapies, APR remains indispensable for patients with low rectal cancer or encroachment on the external anal sphincter (24). Parastomal hernia, one of the most common postoperative complications of APR, has a high incidence of up to 50%, thereby severely affecting the quality of life (2, 3).

Parastomal hernia (PSH) is a type of incisional hernia whose formation is often multifactorial (1). It’s worth noting that the development of incisional hernia may result from tissue trauma and an imbalance between tension and counter-tension created by different forces acting on the anterior abdominal wall (25). It is reported that the development of incision hernia can be divided into patient factors and surgical factors, with patient factors including older age, male sex, obesity, COPD, malnutrition, and previous pelvic radiation; and surgical factors including emergency surgery, surgical approach, size of the surgical site, and use of mesh (26). However, few studies demonstrated the association between the development of PSH and radiological measurements (16, 17).

In the present study, we hypothesized that the CT findings may be associated with the development of PSH in patients who underwent APR. Six radiological factors were significantly associated with the development of PSH in univariate logistic regression analysis, including thickness of SAT, thickness of rectus abdominis, SAT rate, VAT rate, skeletal muscle rate, and colostomy surface area. In the final multivariate analysis, we identified three independent risk factors: thickness of rectus abdominis, SAT percentage, and colostomy surface area. In our study, the incidence of PSH was 44.4%, which is almost congruent with the prevalence of 50% reported in the published literature (2, 3). In addition, the 3-year cumulative incidence rate of PSH was analyzed using Kaplan–Meier curves. We found that high SAT percentage (>median) and large colostomy surface area (>median) were associated with a higher incidence rate than the control group (56.7% vs. 21.5% and 47.3% vs. 33.6%). However, the conclusion was opposite when the thickness of the rectus abdominis was analyzed (36.7% vs. 46.2%).

Several retrospective studies published in recent years evaluated the predictive value of subcutaneous fat for the development of PSH (16, 17, 27). The study of High Subcutaneous Fat Area Is an Independent Risk Factor for Parastomal. Hernia after Transperitoneal Colostomy for Colorectal Cancer indicated that the Subcutaneous fat area was significantly associated with the development of PSH after colostomy, which was consistent with the result in the present study (16). And the conclusion was also coincident with L ele et al’s research (27). However, the association between the subcutaneous fat and PSH was not detected in the study of Clinical and Radiologic Predictors of Parastomal Hernia Development After End Colostomy (17). The subcutaneous adipose tissue content in Jan Pieter’s research was defined as the total volume of extra-abdominal fat between the cranial endplate of the first lumbar vertebra and the cranial part of the pubic bone, which was different from the present study. The stoma is usually placed about the level of the umbilicus in patients who underwent APR surgery (21). Therefore, the SAT content measured at the level of the umbilicus may predict the development of PSH better, which has been verified in Takashi’s study (16).

In the present study, the rectus abdominis thickness measured at the midpoint of the left rectus abdominis muscle on the axial CT slice of the umbilical plane was significantly associated with PSH development. To the best of our knowledge, there is currently no report describing the predictive value of rectus abdominis thickness for PSH in patients with colostomy. The creation of an end colostomy performed in patients included in the present research was through the rectus muscle in the left lower abdomen, and a thicker rectus muscle could better withstand the impact caused by increased intra-abdominal pressure when standing or coughing. R Sjödahl et al. reported that the construction of a colostomy through the rectus abdominis muscle could significantly reduce the risk of PSH (28), of which 130 patients were enrolled and 107 patients underwent colostomy surgery through the rectus abdominis muscle.

To our knowledge, only one study reported the association between colostomy surface area and the development of PSH in the past few years (17). In the present research, the 3-year cumulative incidence rate of PSH increased by 14% in patients whose colostomy surface area was larger than 6.87 cm^2^, and all patients developed PSH after 3 years postoperatively if the stoma area was larger than 10 cm^2^. In addition, Jan Pieter et al. also reported that PSH occurred in all patients with a surface area of the abdominal wall defect larger than 10 cm^2^ (17). Therefore, 10 cm^2^ may be the critical threshold surface area of the abdominal wall defect leading to PSH development, which could provide reference and guidance for surgeons when performing colon stoma construction.

Approximately 30% of the patients diagnosed with PSH may ultimately require surgical repair, due to the development of complications, such as strangulation, incarceration, obstruction, or unsuitable for the ostomy bag mounting (7, 8). It is worth noting that the recurrence of PSH repair surgery is range from 5% to 76% (7, 9, 29). Therefore, the prevention of PSH is important. Recently years, extraperitoneal colostomy has been suggested to be effective to prevent PSH (10, 11). However, the clinical application was limited due to the postoperative complications (12). In the present study, we found that the thickness of the rectus abdominis, the SAT percentage, and the colostomy surface area were significantly associated with the development of PSH. In particular, the thickness of the rectus abdominis and the SAT percentage, as preoperative risk factors, may help surgeons screen out high-risk patients and select appropriate individualized treatment strategies.

The present research has several limitations. First, this is a retrospective research exploring the risk factors for PSH development, and unobserved confounders remained. A prospective observational cohort study would be ideal. Second, the sample size of the present study is still small, because the implementation of abdominoperineal resection has shown a downward trend with the advancement of surgical techniques for the past few years. Even so, three independent risk factors, including the thickness of rectus abdominis, the SAT rate, and the colostomy surface area, were detected in the present research. However, further studies with a large sample size are needed to construct a clinical prediction model for PSH. Third, our study involved only a Chinese population at a single center. Therefore, a prospective and multi-center observational cohort study with a large sample size is required in the future.

## Conclusion

In the present study, we found that the thickness of the rectus abdominis, the SAT percentage, and the colostomy surface area were significantly associated with the development of PSH, which may be potential predictors for PSH.

## Data Availability

The raw data supporting the conclusions of this article will be made available by the authors, without undue reservation.
